# Galectins, Eosinophiles, and Macrophages May Contribute to *Schistosoma japonicum* Egg-Induced Immunopathology in a Mouse Model

**DOI:** 10.3389/fimmu.2020.00146

**Published:** 2020-03-13

**Authors:** Zhanhong Ye, Shiguang Huang, Yanxia Zhang, Xu Mei, Huanqin Zheng, Meiyu Li, Jianhuang Chen, Fangli Lu

**Affiliations:** ^1^Department of Parasitology, Zhongshan School of Medicine, Key Laboratory of Tropical Disease Control of Ministry of Education, Sun Yat-sen University, Guangzhou, China; ^2^School of Stomatology, Jinan University, Guangzhou, China; ^3^Artemisinin Research Center and Institute of Science and Technology, Guangzhou University of Chinese Medicine, Guangzhou, China; ^4^Medical Experimental Teaching Center, Zhongshan School of Medicine, Sun Yat-sen University, Guangzhou, China

**Keywords:** *Schistosoma japonicum*, mice, immunopathology, Gal-1, Gal-3, eosinophils, macrophages

## Abstract

Schistosomiasis is a severe public health problem, which can cause tissue fibrosis and can even be fatal. Previous studies have proven that galectins and different kinds of cells involve in the regulation of tissue fibrosis process. In this study, outbred Kunming mice were infected with *Schistosoma japonicum* (*S. japonicum*). Our results showed that compared with uninfected mice, there were severe egg granulomatous inflammation and tissue fibrosis in the livers, spleens, and large intestines of *S. japonicum*-infected mice at 8 weeks post-infection (p.i.), and the number of eosinophils by hematoxylin and eosin staining and CD68 macrophage-positive area by immunohistochemical staining were significantly increased. Detected by using quantitative real-time reverse transcription-polymerase chain reaction (qRT-PCR), at 8 weeks after *S. japonicum* infection, the mRNA expression levels of galectin (Gal)-1, Gal-3, CD69, eosinophil protein X (EPX), and chitinase 3-like protein 3 (Ym1) were significantly increased in liver, spleen, and large intestine; eotaxin-1 (CCL11) and eosinophil cationic protein were significantly increased in both liver and spleen; eotaxin-2 (CCL24) and Arginase1 (Arg1) were significantly increased in both spleen and large intestine; and CD200R was significantly increased in both liver and large intestine. However, interleukin (IL)-1ß and inducible nitric oxide synthase (iNOS) were only significantly increased in liver. The M2/M1 ratio of CD200R/CD86 genes was significantly increased in liver, and ratios of Ym1/IL-1β and Ym1/iNOS were significantly increased in liver, spleen, and large intestine of *S. japonicum*-infected mice. *Ex vivo* study further confirmed that the levels of Gal-1, Gal-3, CD200R, Arg1, and Ym1 were significantly increased, and the ratios of CD200R/CD86 and Ym1/IL-1β were significantly increased in peritoneal macrophages isolated from *S. japonicum*-infected mice at 8 weeks p.i. In addition, correlation analysis showed that significant positive correlations existed between mRNA levels of Gal-1/Gal-3 and EPX in liver, between Gal-3 and Ym1 in both liver and large intestine, and between Gal-3 and CD200R in peritoneal macrophages of *S. japonicum*-infected mice. Our data suggested that Gal-1, Gal-3, eosinophils, and macrophages are likely involved in the development of egg granulomatous response and fibrosis induced by *S. japonicum* infection.

## Introduction

Schistosomiasis is a zoonotic parasitic disease caused by *Schistosoma* spp. among human beings and animals, which affects nearly 250 million population worldwide (1). Three main species of *Schistosoma*—*Schistosoma japonicum* (*S. japonicum*), *S. mansoni*, and *S. haematobium*—have the most wide prevalence globally. *S. japonicum*, mainly distributed in China, the Philippines, and Indonesia, can cause severe chronic schistosomiasis japonica (2). The eggs of *S. japonicum* immigrate and deposit in liver and intestine tissues, which recruit macrophages, neutrophils, and eosinophils into the tissues during granulomatous reactions (3), and fibrotic deposits distributed around granulomas results in pipestem fibrosis (4). However, so far, the mechanism of tissue fibrosis caused by *S. japonicum* remains not fully understood.

Eosinophils are end effector cells involved in host protection against helminth infection (5). It has been reported that granuloma eosinophils are highly activated and produce the majority of Th2 cytokines in granulomatous inflammation, which may be important determiners of immunopathology in murine schistosomiasis caused by *S. mansoni* (6). The eosinophil granulocytes contain four major proteins, i.e., eosinophil cationnic protein [ECP; (7)], eosinophil peroxidase [EPO; (8)], eosinophil protein X (EPX)/eosinophil-derived neurotoxin (9), and major basic protein [MBP; (10)]. Eotaxin is an essential regulator of eosinophil trafficking during healthy conditions (11) and inflammation (12). EPO and MBP are correlated with cytokine responses of macrophages and CD4^+^ T cells, and mice deficient in either EPO or MBP developed significantly higher worm burdens of *Litomosoides sigmodontis* than wild-type mice (13). In patients infected with *S. haematobium*, both ECP and EPX protein levels are higher in association with rubbery papules in genital lavage, which may serve as markers for a potential early-stage inflammatory lesion in female genital schistosomiasis (14). In addition, increased levels of ECP, EPO, and EPX in sera of patients are related to cystic fibrosis (15). A study demonstrated that primary murine hepatic myofibroblasts derived from granulomas of *S. mansoni*-infected mice can produce interleukin (IL)-5 and eotaxin, which may contribute to maintenance of local eosinophilia in schistosomal hepatic granulomas (16). Eotaxin/eotaxin-1 (CCL11) drives tissue infiltration of eosinophils and mast cells, which can promote pathogenesis in patients with diopathic retroperitoneal fibrosis (17). Thus, eosinophils play an essential role in granulomas and tissue fibrosis.

Macrophages are divided into classically activated macrophages (M1) and alternatively activated macrophages (M2). M1 type macrophages can release monocyte chemotactic protein-1β and inducible nitric oxide synthase (iNOS) and mainly promote inflammatory reaction, whereas M2 type macrophages play a role in the immunoregulation and tissue remodeling (18). It has been reported that hepatitis B virus-mediated liver disease is associated with high level of infiltrated human macrophages with M2-like activation phenotype (19). M2 polarization has been proven to be associated with the process of tissue fibrosis (20). During *S. japonicum* infection, macrophages and schistosome soluble egg antigen (SEA) interaction play a critical role in regulation of host immune responses (21).

Galectins are a family of carbohydrate-binding proteins that are involved in many physiological functions, and 15 members have been identified in various cells and tissues (22). They involve in immunity (23), apoptosis (24), immune tolerance, inflammation (25), and cell adhesion (26). Galectins play important roles in the development of acute inflammation as well as chronic inflammation (27). Both galectin (Gal)-1 and Gal-3 are distributed widely in different cells and tissue types, including innate and adaptive immune cells (23, 28). Gal-1 and Gal-3 facilitate the proliferation of hepatic stellate cells (HSCs) and play an important role in liver fibrosis (29). Some researchers proved that down-regulated expression levels of α-smooth muscle actin (SMA) and transforming growth factor (TGF)-β1 and improved liver fibrosis have been observed in silencing Gal-1 mouse models (30). Macrophage-derived Gal-3 is fundamental for the activation of myofibroblasts (31). *S. mansoni*-infected Gal-3^−/−^ mice had an increase of monocytes and eosinophils in the granulomas from acute and chronic phases of the disease (32). Granuloma-derived stromal cells from *S. mansoni*-infected Lgals3^−/−^ mice express lower levels of α-SMA and eotaxin and higher levels of IL-4 and significant inflammatory infiltration than Lgals3^+/+^ infected mice (33). Gal-3 inhibitor is proven to significantly decrease the percentage of liver and kidney fibrosis area in a non-alcoholic steatohepatitis mouse model (34). However, the concrete mechanism of how galectins influence schistosomiasis fibrosis remains unknown.

To assess the functions of galectins, eosinophils, and macrophages during the stage of *S. japonicum* egg deposition-induced inflammation and fibrosis, in the present study, we compare the expression levels of Gal-1, Gal-3, eosinophil chemoattractant (CCL11 and eotaxin-2 [CCL24]), eosinophil marker (CD69), eosinophil granule proteins (ECP and EPX), M1 macrophage markers (CD86, IL-1β, and iNOS), and M2 macrophage markers (CD200R, Arginase1 [Arg1], and chitinase 3-like protein 3 [Ym1]) in the livers, spleens, large intestines, and peritoneal macrophages of mice with chronic schistosomiasis japonica. Based on the relationship among galectins, eosinophils, macrophage polarization, and pathology of schistosomiasis japonica, our data demonstrated that Gal-1, Gal-3, eosinophils, and macrophages play important roles in *S. japonicum* egg deposition-induced immune response and fibrosis in advanced schistosomiasis japonica mouse model.

## Materials and Methods

### Ethics Statement

*In vivo* experiments were approved by the Animal Experimentation Ethics Committee of Zhongshan School of Medicine on Laboratory Animal Care at Sun Yat-sen University (No. 2016-081) and were carried out in strict accordance with institutional Guidelines for Care and Use of Laboratory Animals.

### Mice and Parasite Infection

Female Kunming mice (outbred, 6–8 weeks old) were purchased from the Animal Facility of Sun Yat-sen University. *Oncomelania hupensis* snails were obtained from the National Institute of Parasitic Diseases, Chinese Center for Disease Control and Prevention (Shanghai, China). Forty-six mice were used in this experiment, which were divided into two groups, i.e., a naive group and an infected group, each containing 23 mice. Infected mice were infected percutaneously with 30 cercariae of *S. japonicum*.

### Histopathology

At 8 weeks post-infection (p.i.), naive mice and *S. japonicum*-infected mice were euthanatized by CO_2_ asphyxiation and their livers, spleens, and large intestines were harvested. Samples were fixed in 10% buffered natural formaldehyde (Guangzhou Chemical Reagent Factory, China) for over 48 h. The paraffin-embedded tissues from each mouse were sectioned at 4 μm and prepared for hematoxylin and eosin (H&E) staining (Sigma-Aldrich, Shanghai, China). The histopathological changes of liver, spleen, and large intestine from each mouse were determined under 200×, 400×, or 1000× magnification in three noncontiguous sections. The number of eosinophils were quantified using images captured with a digital camera system under 1000 × magnification and analyzed by using Image-Pro Plus (Image Z1 software, version 6.0, Media Cybernetics, MD, United States), and the density of eosinophils was expressed as the number of eosinophils per square millimeter.

### Sirius Red Staining

To detect the deposition of collagen fiber from different tissues, paraffin-embedded liver, spleen, and large intestine from each mouse were sectioned at 4 μm and stained by Sirius red stain kit (Beijing Leagene Biotchnology Co., Ltd., China).

### Immunohistochemical Staining

The paraffin-embedded liver, spleen, and large intestine sections (4-μm) were deparaffinized and rehydrated in distilled water. Heat-induced antigen retrieval was carried out in an 800-W microwave oven for 30 min. Sections were treated with 3% hydrogen peroxide in methanol for 10 min at 37°C and then incubated in 10% normal goat serum with 1% bovine serum albumin (Sigma-Aldrich) in PBS (pH 7.4) for 10 min at room temperature to block non-specific binding. After washing with PBS, sections were incubated with rabbit anti-Gal-1 (1:500 dilution; Wuhan Boster Biological Engineering Co., Ltd., Wuhan, China), rabbit anti-Gal-3 polyclonal antibody (IgG1; 1:200 dilution; Bioss, Beijing, China), or rabbit anti-CD68 (1:200 dilution; Wuhan Boster Biological Engineering Co., Ltd.) overnight at 4°C. Those sections incubated with secondary antibodies alone were used as isotype controls. Immunohistochemical staining was then performed with a streptavidin–biotin–peroxidase complex kit and developed with diaminobenzidine tetrahydrochloride (Beijing Zhongshan Golden Bridge Biotechnology, Beijing, China). The sections were counterstained with hematoxylin and positive cells were identified by dark-brown staining under light microscopy. The immunohistochemistry signal (positive areas) of CD68 were quantified using images captured with a digital camera system under 400× magnification and analyzed by using Image-Pro Plus (Image Z1 software, version 6.0, Media Cybernetics, MD, United States).

### Isolation of Murine Peritoneal Macrophages

At 8 weeks p.i., eight naive mice and eight *S. japonicum*-infected mice were injected intraperitoneally with 2 ml 3% thioglycollate broth (Sigma-Aldrich) solution in PBS once daily for 3 days, and animals were sacrificed and their peritoneal lavage fluid were spun at 800 g at 4°C for 5 min, and the pelleted peritoneal macrophages were resuspended and seeded at 5 × 10^5^ cells/well in 12-well plates (Corning, NY, United States). After 4 h at 37°C in a 5% CO_2_ atmosphere, cells were washed and collected. Samples were stored at −80°C until subjected to further analysis.

### RNA Isolation, cDNA Synthesis, and Quantitative Real-Time Reverse Transcription-Polymerase Chain Reaction (qRT-PCR)

Total RNA was extracted from about 100 mg of mouse liver, spleen, and large intestine tissues of each mouse or peritoneal macrophages isolated from each mouse using an RNA Extraction Kit (Takara Bio Inc., Shiga, Japan). The quality and quantity of RNA were determined by NanoDrop 2000 spectrophotometer (Thermo Fisher, Waltham, MA, United States). First-strand cDNA was constructed from 1.0 μg of total RNA with oligo(dT) as primers using a PrimeScript 1st Strand cDNA Synthesis Kit (Takara Bio Inc.). The primer sequences are listed in Table 1. To determine mRNA levels of CD69, CCL11, CCL24, ECP, and EPX in liver, spleen, and large intestine tissues and mRNA levels of CD86, CD200R, Gal-1, Gal-3, Arg1, Ym1, IL-1β, and iNOS in liver, spleen, and large intestine tissues, and peritoneal macrophages, qRT-PCR measurements were performed using SYBR Green QPCR Master Mix (Takara Bio Inc.). Briefly, a total of 10 μl reaction mixture contained 5.0 μl of SYBR® Premix Ex TaqTM (2×), 0.5 μl of each primer (10 pM), 3.0 μl of dH_2_O, and 1.0 μl of cDNA (0.2 μg/μl). Amplification was pre-denaturized for 30 s at 95°C, followed by 43 cycles of 5 s at 95°C and 20 s at 60°C with a CFX96 real-time PCR detection system (Bio-Rad Laboratories, Hercules, CA, United States). The mRNA expression levels of CD69, CD86, CD200R, galectins, cytokines, and chemokines were normalized to that of mouse housekeeping gene, GAPDH. The results were expressed as fold change compared with uninfected mice.

**Table 1:** Primer sequences of genes used for quantitative real-time reverse transcription-polymerase chain reaction assays.

**Genes**	**Forward primer (5**′** → 3**′**)**	**Reverse primer (5**′** → 3**′**)**	**Accession**
GAPDH	ACTCCACTCACGGCAAATTC	TCTCCATGGTGGTGAAGACA	NM_001289726.1
IL-1β	AATGACCTGTTCTTTGAAGTTGA	TGATGTGCTGCTGCGAGATTTGAAG	NM_008361.4
iNOS	GTTCTCAGCCCAACAATACAAGA	GTGGACGGGTCGATGTCAC	NM_010927.4
Ym1	AGAAGGGAGTTTCAAACCTGGT	GTCTTGCTCATGTGTGTAAGTGA	NM_009892.3
Arg1	CAGAAGAATGGAAGAGTCAG	CAGATATGCAGGGAGTCACC	NM_007482.3
Gal-1	CGCCAGCAACCTGAATC	GTCCCATCTTCCTTGGTGTTA	NM_008495.2
Gal-3	AACACGAAGCAGGACAATAACTGG	GCAGTAGGTGAGCATCGTTGAC	NM_010705.3
ECP	ATCCAAGTGGCTTGTGCAGTGAC	TAAGGTGTTCTCCTCCGACTGGTG	XM_021155370.1
EPX	CACGAACCAGCGATTCCAGGAC	GCTCAGCGGCTAGGCGATTG	NM_007946.2
CCL11	CAGATGCACCCTGAAAGCCA	CAGGTGCTTTGTGGCATCCT	NM_011330.3
CCL24	CTCAGATGGTGGTGTGCTTGCC	TCCTCTTGCCTCTGCCTTCTGG	NM__019577.5
CD69	GGGCTGTGTTAATAGTGGTCCTC	CTTGCAGGTAGCAACATGGTGG	NM_001033122
CD86	ACGTATTGGAAGGAGATTACAGCT	TCTGTCAGCGTTACTATCCCGC	NM_019388
CD200R	TGTGAGACAGTAACACCTGAAGG	TGCCATTGCCTCACAGACTGCA	NM_021325

### Statistical Analysis

Statistical analysis was performed using SPSS Statistics version 22.0 (SPSS Inc., Chicago, IL, United States). Data are presented as means ± standard deviation (SD) with at least three independent biological replicates. Student *t*-test was used to compare data between different groups. Pearson's correlation coefficient was used to analyze correlations between the levels of CD69, CD86, CD200R, galectins, cytokines, and chemokines. *P* < 0.05 was considered statistically significant.

## Results

### The Pathology and Fibrosis in the Livers, Spleens, and Large Intestines of Mice Infected With *S. japonicum*

**Figure 1:**
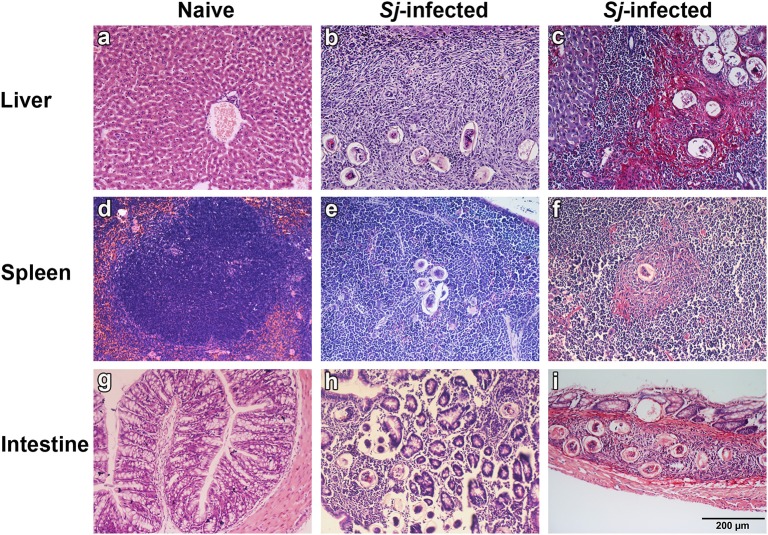
Histopathological changes and fibrosis in the liver, spleen, and large intestine tissues of *S. japonicum*-infected mice at 8 weeks p.i. No histological change was observed in the liver, spleen, and large intestine tissues of uninfected mice **(a,d,g)**. Egg granulomas **(b,e,h)** and collagen deposition **(c,f,i)** were observed in the liver, spleen, and large intestine tissues of *S. japonicum*-infected mice. There were four mice in each group. Original magnification 200× (scale bar = 200 μm); H&E stain **(a,b,d,e,g,h)** and Sirius red stain **(c,f,i)**.

The livers, spleens, and large intestines of both naive and *S. japonicum*-infected mice were examined histologically at 8 weeks p.i. The results showed that the sections of livers, spleens, and large intestines from uninfected control mice were negative for pathological changes and egg granulomas. However, the sections from infected mice showed severe histological change and *S. japonicum* egg granulomas with eosinophils and other inflammation cells gathering in the tissues of liver, spleen, and large intestine. Classical pigmented Kupffer cells were found around liver granulomas (Figures 1a,b,d,e,g,h). Sirius red staining showed that large amount of collagen deposition was observed around granulomas in liver, spleen, and large intestine tissues of *S. japonicum*-infected mice (Figures 1c,f,i).

### Eosinophils in the Livers, Spleens, and Large Intestines of *S. japonicum*-Infected Mice

**Figure 2:**
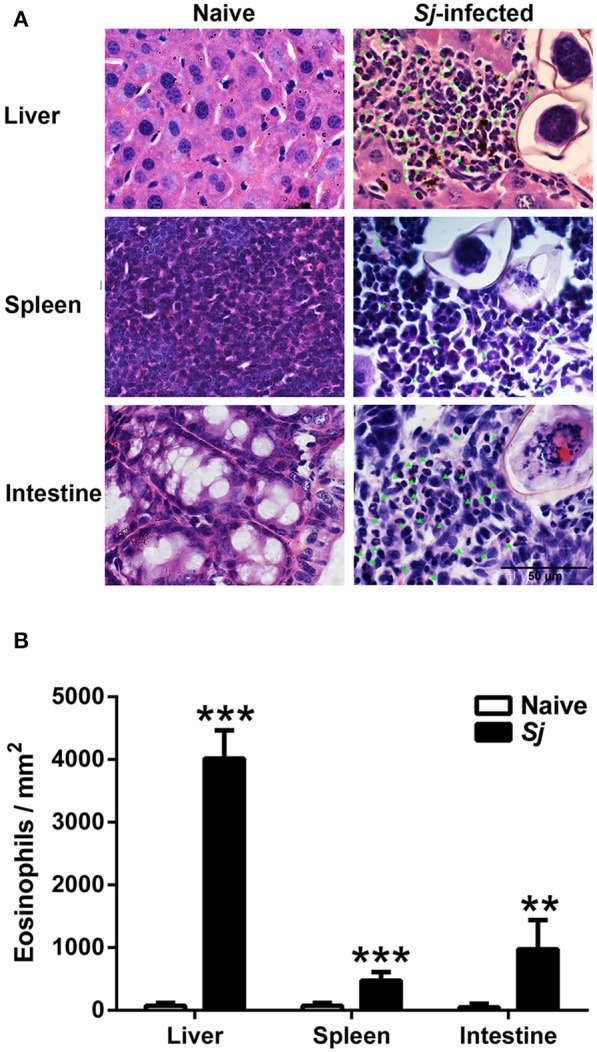
Observation of eosinophils around egg granulomas **(A)** and eosinophil count **(B)** in liver, spleen, and large intestine tissues of *S. japonicum*-infected mice at 8 weeks p.i. Eosinophils were indicated by green arrows. Original magnification 1,000× (scale bar = 50 μm); H&E stain. Data are presented as means ± SD; there were four mice in each group. **P < 0.01 and ***P < 0.001, *S*. *japonicum*-infected mice vs. naive mice.

By H&E staining, compared with uninfected controls, there was significantly increased eosinophil infiltrate around egg granulomas at 8 weeks p.i. (Figure 2A). Quantitative analysis showed that the number of eosinophils were significantly increased in the livers (*P* < 0.001), spleens (*P* < 0.001), and large intestines (*P* < 0.01) of *S*. *japonicum*-infected mice compared with uninfected controls (Figure 2B).

### The mRNA Levels of CD69, CCL11, CCL24, ECP, and EPX in the Livers, Spleens, and Large Intestines of *S. japonicum*-Infected Mice

**Figure 3:**
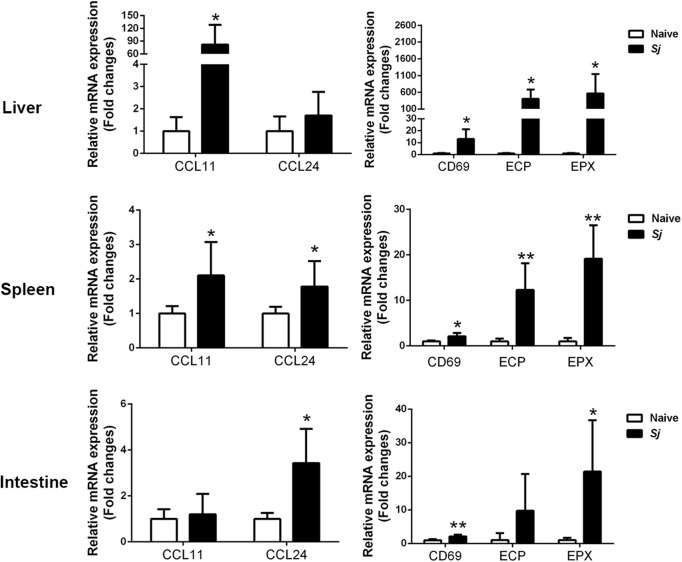
The mRNA expressions of CCL11, CCL24, CD69, EPX, and ECP in the livers, spleens, and large intestines of *S. japonicum*-infected mice at 8 weeks p.i. Values are means from triplicate measurements and data are presented as means ± SD; there were eight mice in each group, and the data represent two experiments. *P < 0.05 and **P < 0.01, *S*. *japonicum*-infected mice vs. naive mice.

Compared with uninfected controls, *S. japonicum-*infected mice presented significantly elevated mRNA expression levels of CCL11, ECP, EPX, and CD69 in the livers (*P* < 0.05); significantly elevated levels of CCL11 (*P* < 0.05), CCL24 (*P* < 0.05), CD69 (*P* < 0.05), EPX (*P* < 0.01), and ECP (*P* < 0.01) in the spleens; and significantly elevated levels of CCL24 (*P* < 0.05), EPX (*P* < 0.05), and CD69 (*P* < 0.01) in the large intestines at 8 weeks p.i. (Figure 3).

### Immunohistochemical Staining and mRNA Expression of Gal-1 and Gal-3 in the Livers, Spleens, and Large Intestines of *S. japonicum*-Infected Mice

**Figure 4:**
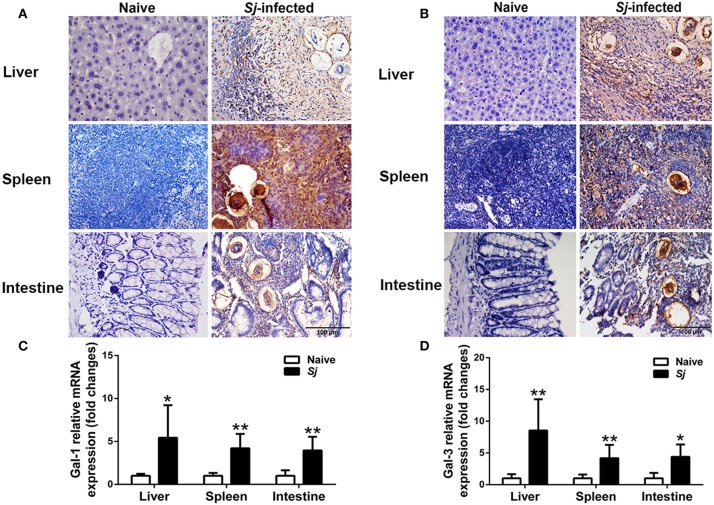
Immunohistochemistry for Gal-1 **(A)** and Gal-3 **(B)** and mRNA expressions of Gal-1 **(C)** and Gal-3 **(D)** in the livers, spleens, and large intestines of *S. japonicum-*infected mice at 8 weeks p.i. Original magnification 400× (scale bar = 100 μm). There were four mice for immunohistochemistry analysis. For mRNA expression analysis, values are means from triplicate measurements and data are presented as means ± SD; there were eight mice in each group and the data represents from two experiments. *P < 0.05 and **P < 0.01, *S*. *japonicum*-infected mice vs. naive mice.

There were only a few Gal-1- and Gal-3-positive cells observed in the liver, spleen, and large intestine of uninfected mice. However, a large amount of Gal-1 and Gal-3 positive cells labeled dark brown, especially around the granulomas, were presented in the liver, spleen, and large intestine of *S. japonicum-*infected mice (Figures 4A,B). The Gal-1 and Gal-3 mRNA expression levels in the liver, spleen, and large intestine tissues were measured. Compared with uninfected controls, *S. japonicum-*infected mice presented significantly elevated Gal-1 and Gal-3 levels in the livers (*P* < 0.05 and *P* < 0.01, respectively), spleens (*P* < 0.01), and large intestines (*P* < 0.01 and *P* < 0.05, respectively) of *S. japonicum-*infected mice at 8 weeks p.i. (Figures 4C,D).

### Immunohistochemical Staining for CD68^+^ Macrophages and mRNA Expression Levels of CD86, CD200R, IL-1β, iNOS, Arg1, and Ym1 in the Livers, Spleens, and Large Intestines of *S. japonicum*-Infected Mice

**Figure 5:**
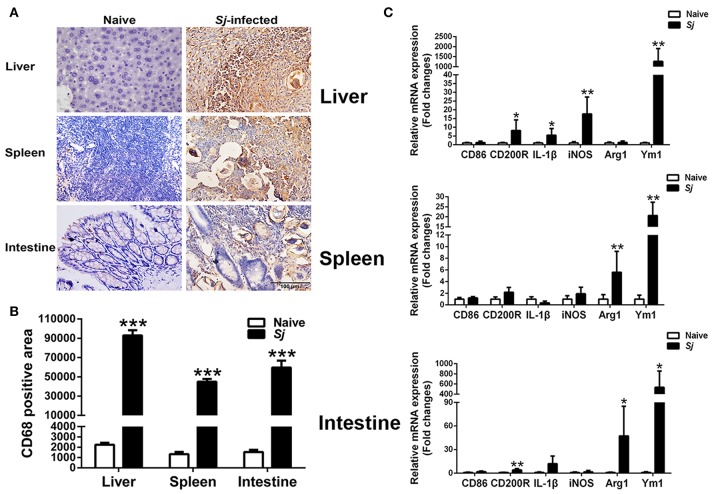
Immunohistochemistry for CD68^+^ macrophages **(A)**, quantitative analysis of the positive areas **(B)**, and mRNA expressions of CD86, CD200R, IL-1β, iNOS, Arg1, and Ym1 **(C)** in the livers, spleens, and large intestines of *S. japonicum*-infected mice at 8 weeks p.i. Original magnification 400× (scale bar = 100 μm). There were four mice for immunohistochemistry analysis. For mRNA expression analysis, values are means from triplicate measurements and data are presented as means ± SD; there were eight mice in each group and the data represents from two experiments. *P < 0.05, **P < 0.01, and ***P < 0.001, *S*. *japonicum*-infected mice vs. naive mice.

Detected by immunohistochemical staining, compared with uninfected controls, there were more CD68^+^ macrophages around egg granulomas in the livers, spleens, and large intestines of *S. japonicum*-infected mice (Figure 5A). Quantitative analysis showed significantly higher CD68 macrophage-positive area in the livers, spleens, and large intestines of *S. japonicum*-infected mice (*P* < 0.001) in comparison with uninfected controls (Figure 5B). In addition, compared with uninfected controls, *S. japonicum*-infected mice presented significantly elevated mRNA levels of CD200R (*P* < 0.05), IL-1β (*P* < 0.05), iNOS (*P* < 0.01), and Ym1 (*P* < 0.01) in the livers; elevated levels of Arg1 and Ym1 in the spleens (*P* < 0.01); and elevated levels of CD200R (*P* < 0.01), Arg1 (*P* < 0.05), and Ym1 (*P* < 0.05) in the large intestines at 8 weeks p.i. (Figure 5C).

### The mRNA Expression Levels of CD86, CD200R, Gal-1, Gal-3, IL-1β, iNOS, Arg1, and Ym1 in Peritoneal Macrophages of *S. japonicum*-Infected Mice

**Figure 6:**
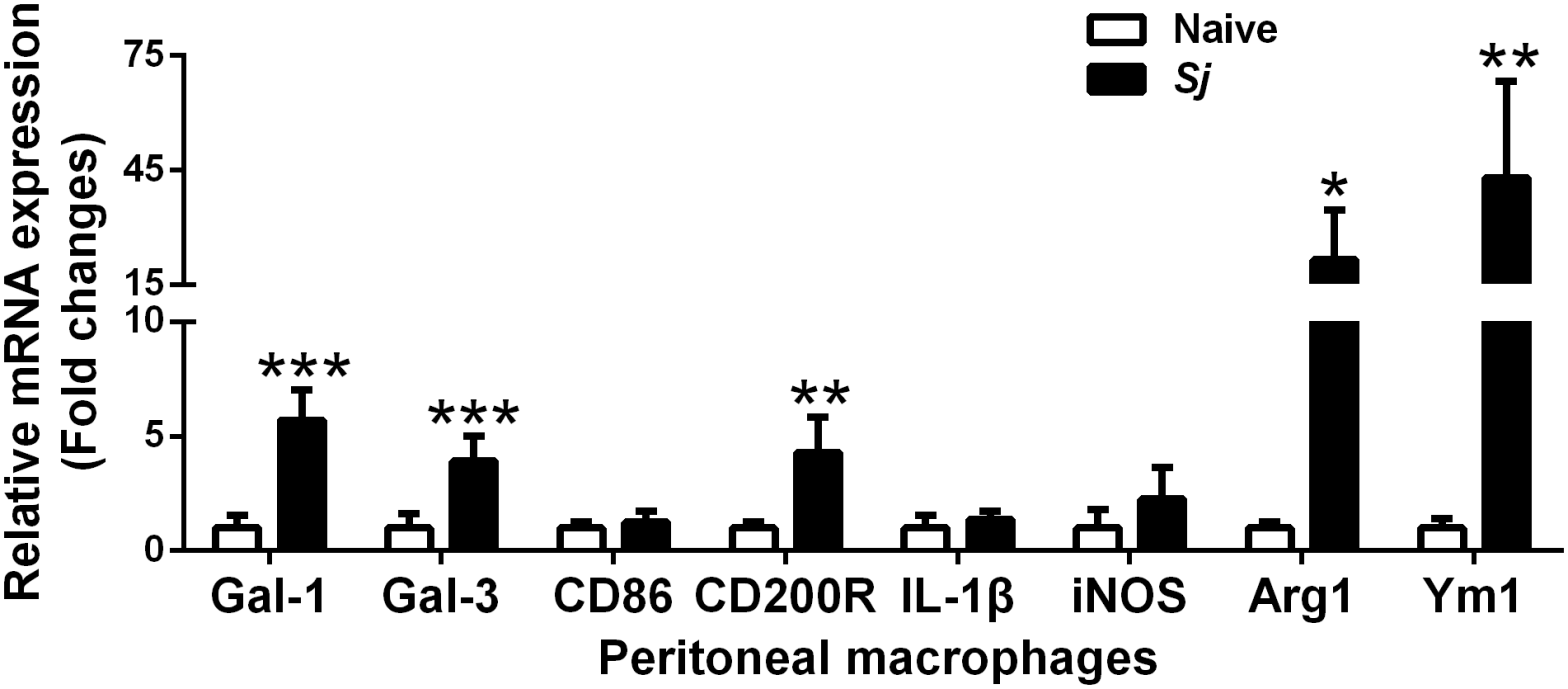
The mRNA expressions of Gal-1, Gal-3, CD86, CD200R, IL-1β, iNOS, Arg1, and Ym1 in peritoneal macrophages of *S. japonicum*-infected mice at 8 weeks p.i. Values are means from triplicate measurements and data are presented as means ± SD; there were eight mice in each group and the data represents from two experiments. *P < 0.05, **P < 0.01, and ***P < 0.001, *S*. *japonicum*-infected mice vs. naive mice.

Compared with uninfected controls, there were significantly elevated mRNA levels of CD200R (*P* < 0.01), Gal-1 (*P* < 0.001), Gal-3 (*P* < 0.001), Arg1 (*P* < 0.05), and Ym1 (*P* < 0.01) in the peritoneal macrophages isolated from *S. japonicum*-infected mice at 8 weeks p.i. (Figure 6).

### The M2/M1 Ratios of CD200R/CD86, Ym1/IL-1β, and Ym1/iNOS in the Livers, Spleens, Large Intestines, and Peritoneal Macrophages of *S. japonicum*-Infected Mice

**Figure 7:**
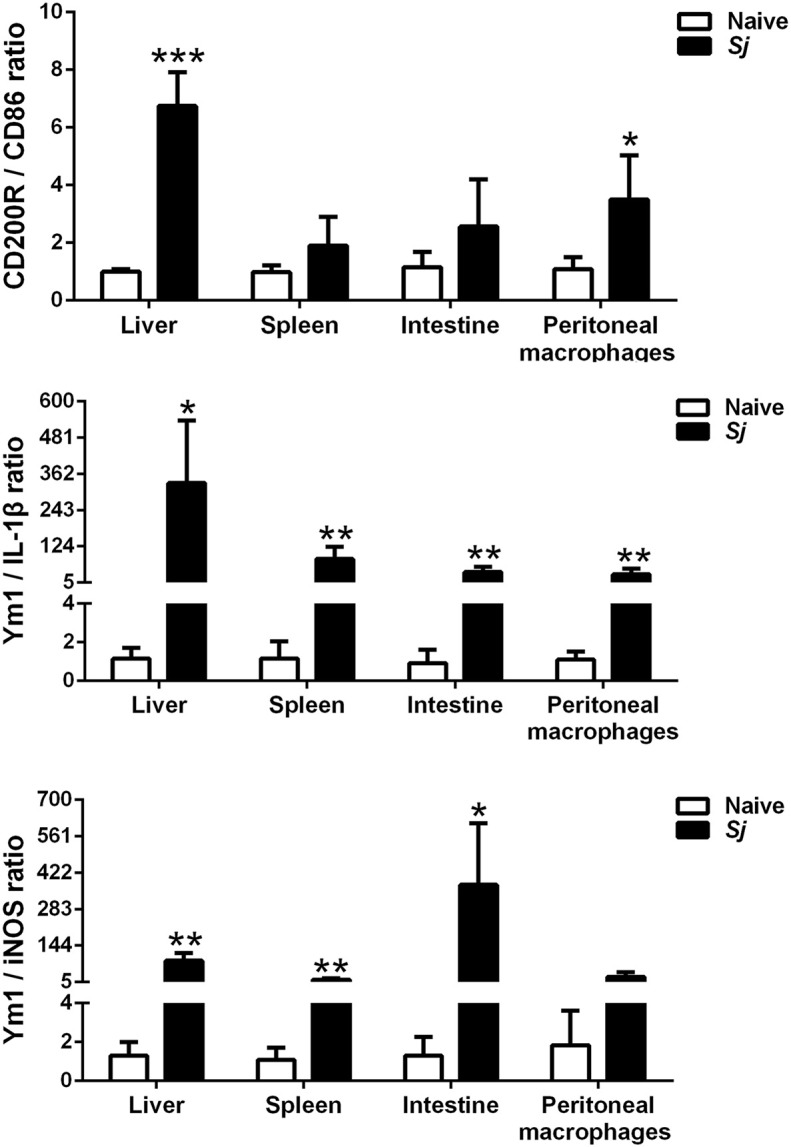
The ratios of M2/M1 gene expression (CD200R/CD86, Ym1/IL-1β, and Ym1/iNOS) in the livers, spleens, large intestines, and peritoneal macrophages of *S. japonicum*-infected mice at 8 weeks p.i. Data are presented as means ± SD; there were eight mice in each group and the data represents from two experiments. *P < 0.05, **P < 0.01, and ***P < 0.001, *S*. *japonicum*-infected mice vs. naive mice.

Compared with uninfected controls, *S. japonicum*-infected mice presented significantly elevated M2/M1 ratios of CD200R/CD86 (*P* < 0.001), Ym1/IL-1β (*P* < 0.05), and Ym1/iNOS (*P* < 0.01) in the liver; elevated M2/M1 ratios of Ym1/IL-1β and Ym1/iNOS in the spleen (*P* < 0.01); and elevated M2/M1 ratios of Ym1/IL-1β (*P* < 0.01) and Ym1/iNOS (*P* < 0.05) in the large intestine at 8 weeks p.i. Furthermore, there were significantly increased M2/M1 ratios of CD200R/CD86 (*P* < 0.05) and Ym1/IL-1β (*P* < 0.01) in the peritoneal macrophages isolated from *S. japonicum*-infected mice at 8 weeks p.i. (Figure 7).

### Correlation Analysis Between mRNA Expressions of Gal-1, Gal-3, EPX, and Ym1 in the Livers, Spleens, Large Intestines, and Peritoneal Macrophages of *S. japonicum-*Infected Mice

**Figure 8:**
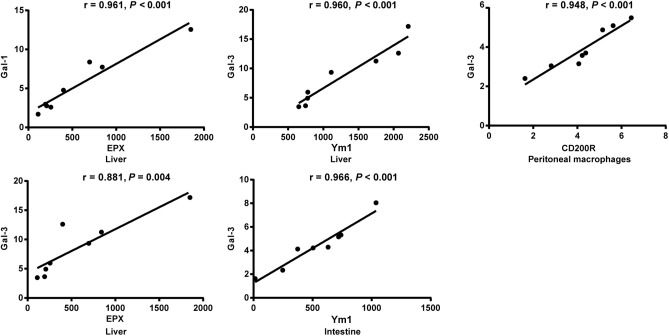
Correlation analysis between the mRNA expressions of Gal-1/Gal-3 and EPX, Ym1, and CD200R in the livers, large intestines, or peritoneal macrophages of *S. japonicum*-infected mice at 8 weeks p.i. The *r* value generates the theoretical line of best fit, and the *P* value indicates the significance of the correlation. There were eight mice in each group and the data represents from two experiments.

The correlations between mRNA levels of different genes in the livers, spleens, large intestines, and peritoneal macrophages of *S. japonicum*-infected mice were analyzed. Only significant correlations were shown here. There were positive and significant correlations between Gal-1 and EPX (*r* = 0.961, *P* < 0.001) and between Gal-3 and EPX (*r* = 0.881, *P* = 0.004) in the livers, between Gal-3 and Ym1 in both the livers (*r* = 0.960, *P* < 0.001) and large intestines (*r* = 0.966, *P* < 0.001), and between Gal-3 and CD200R in the peritoneal macrophages (*r* = 0.948, *P* < 0.001) of *S. japonicum*-infected mice at 8 weeks p.i. (Figure 8).

## Discussion

Schistosomiasis is a chronic and potentially lethal disease, and the disease morbidity is caused by schistosome eggs and not directly by adult worms (35). Liver fibrosis caused by *S. mansoni* and *S*. *japonicum* is one of the most severe complications in advanced schistosomiasis, which usually accompanies portal hypertension and hepatosplenomegaly, and sometimes it is fatal (36). *S. japonicum* eggs can be deposited in ectopic locations, almost all organs and tissues of human host can be involved, and ectopic clinical manifestations and damage have been more frequently reported in patients with cerebrospinal and pulmonary schistosomiasis (37). So far, the understanding of host responses during *S. japonicum* infection remains inadequate. To address this scientific question, we used outbred Kunming mice to develop a mouse model of schistosomiasis japonica, which could thereby allow us to investigate the immune response of tissue pathology and consequent fibrosis caused by *S. japonicum* egg deposition.

Fibrosis is a common pathological outcome of most chronic inflammatory diseases, and both innate and adaptive immune responses contribute to the pathogenesis of fibrosis (38). Eosinophils participate in both innate and adaptive immune response to parasite through antibody-dependent cellular cytotoxicity and antigen-presenting function (5). The granules of eosinophils involve in the effect of anti-parasite immunity, and ECP is important granule protein that acts as toxic to helminths (39, 40). During *S. mansoni* infection, an intense proliferation of eosinophils occurred in hepatic granuloma (41). Eosinophils have the role of destruction of *S. mansoni* eggs *in vivo* (42). *S. haematobium* egg-injected bladders of mice accumulate an eosinophil- and neutrophil-dominated mixed inflammatory infiltrate, and develop fibrosis and urinary dysfunction (43). Eosinophil-deficient mice present markedly ameliorated radiation-induced small intestinal fibrosis, suggesting eosinophils as a crucial factor in the pathogenesis of radiation-induced intestinal fibrosis (44). In the present study, increased eosinophil infiltrations were observed in the granulomas in livers, spleens, and large intestines, which is the histologic hallmark of *S. japonicum* infection. Therefore, we addressed the question of whether egg granuloma can promote eosinophil degranulation. We focused on the expressions of CD69, CCL11, CCL24, ECP, and EPO in livers, spleens, and large intestines. Our data showed that after *S. japonicum* infection, a large number of eosinophils around granulomas in the livers, spleens, and large intestines of mice with advanced schistosomiasis japonica, as expected, the levels of CCL11 and CCL24 were increased in the liver, spleen, or large intestine. In addition, we found that the levels of CD69, ECP, and EPX were significantly increased in the livers, spleen, or large intestines, which indicated that eosinophil degranulation plays a crucial role in the development of egg granuloma formation and fibrosis induced by *S. japonicum* infection.

The pathogenesis of fibrosis is tightly regulated by macrophages in liver, lung, and gut (45). Macrophages produce inflammatory factors such as TGF-β and platelet-derived growth factor to activate HSCs and eventually lead to liver fibrosis, and macrophage polarization is associated with regulation of liver fibrosis (31). In an experiment, researchers combined a mouse model of urogenital schistosomiasis with macrophage depletion, and it has been demonstrated that macrophages modulate acute pathophysiology of *S. haematobium* egg-exposed bladder and necessary for the bladder fibrotic response to *S. haematobium* eggs (46). In the present study, our results showed that compared with uninfected mice, M1 macrophage markers (IL-1ß and iNOS) were only increased in liver; however, M2 macrophage markers (CD200R, Arg1, and Ym1) were increased in liver, spleen, large intestine, or peritoneal macrophages of mice with *S. japonicum* infection. In addition, the M2/M1 ratio of CD200R/CD86 in liver and peritoneal macrophages; the ratios of Ym1/IL-1β and Ym1/iNOS in liver, spleen, and large intestine; and the ratio of Ym1/IL-1β in peritoneal macrophages were significantly increased in *S. japonicum*-infected mice at 8 weeks p.i. Schistosome worm antigen of *S. japonicum* facilitates the generation of M1 macrophages, whereas SEA preferentially promotes M2-polarized phenotype (47). It has been reported that peritoneal cavity is a secondary site of *S. mansoni*-associated inflammation, although it is not in direct contact with the parasite (48, 49). In mammals, tissue-resident macrophages are found all over the body in all organs and serous membranes, including the peritoneal cavity (50, 51). By detecting peritoneal macrophages from *S. mansoni*-infected mice, our data further demonstrated that M2 macrophage polarization plays a predominant role during murine chronic schistosomiasis japonica.

Galectins have been proven to modulate host responses against parasitic infection (52). Studies showed that Gal-1 knockdown can attenuate TGF-β1-induced fibroblast activation *in vitro* (53) as well as reduce macrophages' polarization transform from M1 (pro-inflammatory) to M2 (anti-inflammatory) during glioblastoma multiforme progression in a mouse model (54). Cell-surface glycans and Gal-1 promote HSC activation and migration; therefore, disrupting glycosylation-dependent Gal-1/neuropilin-1 interactions may be a possible therapy for liver fibrosis (55). Gal-3 expression is up-regulated in human liver fibrosis and plays a critical role in liver fibrosis; animal study further demonstrated that myofibroblast activation is Gal-3 dependent (56). Use of Gal-3 inhibitor to cure liver fibrosis caused by non-alcoholic steatohepatitis has satisfied effect (34). Gal-3 directly binds SEA, and high levels of Gal-3 can be found in the granulomas surrounding eggs of *S. mansoni*-infected hamsters (57). In the present study, our data demonstrated that both Gal-1 and Gal-3 were significantly increased in the livers, spleens, large intestines, and peritoneal macrophages of *S. japonicum*-infected mice at 8 weeks p.i., indicating that Gal-1 and Gal-3 play a role in the regulation of egg granuloma pathology during schistosomiasis japonica. Interestingly, we found significant correlations existed between Gal-1 and EPX and between Gal-3 and EPX in the liver, between Gal-3 and Ym1 in both the liver and large intestines, and between Gal-3 and CD200R in the peritoneal macrophages of mice suffered from schistosomiasis japonica. It has been found that Gal-3 is necessary for myofibroblast activation and fibrotic granulomas in *S. mansoni-*infected mice, and inflammatory infiltrate is amplified in Lgals3^−/−^-infected mice in comparison with Lgals3^+/+^-infected mice (33). The number of macrophages is decreased, and smaller granulomas and less collagen fibers are observed in the liver of Gal-3^−/−^ mice infected with *S. mansoni* compared to infected wild-type mice, suggesting a direct correlation of Gal-3 with the fibrosis (32). Thus, Gal-1 and Gal-3 might be involved in egg granuloma fibrosis by regulating eosinophil degranulation and M2 macrophage polarization in advanced schistosomiasis japonica in the mouse model.

In conclusion, egg granuloma and fibrosis play very crucial roles in the pathological process induced by *S. japonicum* infection. Our study has proven that Gal-1, Gal-3, eosinophil and its granule proteins (ECP and EPX), and M2 macrophage may all play crucial roles in regulation of chronic immunopathology in murine schistosomiasis japonica. However, whether Gal-1 and Gal-3 contribute directly or indirectly to modulate *S. japonicum*-induced immune reaction and fibrosis through regulation of eosinophil degranulation and macrophages M2 polarization remains to be further determined.

## Data Availability Statement

The raw data supporting the conclusions of this article will be made available by the authors, without undue reservation, to any qualified researcher.

## Ethics Statement

The animal study was reviewed and approved by the Animal Experimentation Ethics Committee of Zhongshan School of Medicine on Laboratory Animal Care at Sun Yat-sen University (No. 2016-081), and were carried out in strict accordance with institutional Guidelines for Care and Use of Laboratory Animals.

## Author Contributions

FL conceived and designed the experiments, analyzed the data, and wrote the manuscript. ZY performed the experiments and analyzed the data. SH edited the manuscript. YZ participated in analyzing the data. XM, HZ, ML, and JC participated in conducting the experiments.

### Conflict of Interest

The authors declare that the research was conducted in the absence of any commercial or financial relationships that could be construed as a potential conflict of interest.
